# ASTMH – A Means to What End?‡ *The Nexus of Tropical Medicine and Human Rights*

**DOI:** 10.4269/ajtmh.22-0721

**Published:** 2023-01-16

**Authors:** Daniel G. Bausch

**Affiliations:** FIND, Geneva, Switzerland

## INTRODUCTION

Today I want to talk about the nexus of our field of tropical medicine and human rights. As Lina Moses explained in her kind introduction, I’ve been lucky to have very diverse experiences and the opportunity to learn from colleagues and friends in diverse disciplines throughout my career. These experiences include things like setting up a program for Lassa fever in Sierra Leone, running an Ebola treatment unit in Uganda, conducting contact tracing for SARS-CoV-1 in Vietnam, and trapping bats in the Congo to discover the animal reservoir for Marburg virus. The latter, incidentally, is when my family, who is entirely non-scientific, gave up trying to understand what I do for a living. They had more the idea of a white-coat–wearing “let me take your blood pressure” doctor than a bat-trapping doctor in the Congo. My mother eventually gave up, saying, “My son is a doctor, but we don’t like to talk about it…”.

My first exposure to human rights challenges in these settings came in the 1980s when I was at university and medical school in Chicago. It was an era when wars raged in many countries in Central America. I became president of a small chapter of the human rights organization Amnesty International at the Loyola Stritch School of Medicine outside of Chicago. One of our major endeavors related to the Sanctuary Movement—providing shelter and care to refugees, some documented and some not, fleeing the violence in Central America. This subsequently led to further involvement during residency. I started to spend what free time I had in El Salvador supporting various health and human rights projects. These were mostly oriented toward providing avenues for dialogue and free speech about health and supporting young community health workers, or “promotoras de salud,” at a time when the idea of communities banding together was considered leftist, and subversive, by El Salvador’s totalitarian government — the type of idea that could easily get someone “disappeared” in the night from a visit by government death squads. It was clear, very early on, that a definition of health as simply the absence of disease was not equating to a healthy life for most Salvadorans. The complicated social and political roots of their problems, impacting both physical and mental health, could not be ignored.

During my time in El Salvador, the wave of cholera, reintroduced into the Americas in Peru in 1991 and then sneaking its way northward, crashed on the shores of El Salvador, causing major suffering in a country still struggling to emerge from a devastating civil war. There were hundreds of patients in need of care per day. It was my first time working in an isolation and treatment center — this one specifically for cholera but somewhat akin to what I would do years later for Ebola and Marburg. Now, the connection between tropical diseases and the underlying socio-political context and conditions was also becoming clear. Not surprisingly, a few years later, after joining our Society, my first formal contribution to the ASTMH program, in 2004, was a symposium entitled “Social and Political Issues in Tropical Medicine.”

Although not by design, my time in El Salvador began a steady continuum of engagement in scientific and public health projects in low-resource countries experiencing civil unrest and political instability, and the infectious diseases that went with them – Cholera in El Salvador, Lassa fever in Sierra Leone, Marburg hemorrhagic fever in the Democratic Republic of the Congo, and Ebola in Uganda – all countries that, at the time I was there, had beleaguered populations and decimated healthcare systems from years of war and civil unrest. Existential crises seemed like the norm. Incidentally, although not due to war, in 2005 I would go on to experience my own existential crisis, somewhat akin to what war produces, when Hurricane Katrina devastated my then home city of New Orleans. As Lina and many of my Tulane colleagues in the room can attest, it definitely did NOT seem like the norm to us when we were the target.

Experience with the string of the aforementioned largely zoonotic diseases also opened my eyes to the interrelation between human health, animal health, and the environment – what we would today call, a One Health approach.

### The nexus of tropical medicine and human rights.

You don’t have to be around such settings very long to understand that major challenges extend far beyond your particular and often singular disease focus. How to talk to a man who has recently had a limb amputated by child soldiers in Sierra Leone and tell him he should take care to not catch the rodent-borne disease, Lassa fever? Or to explain to a mother in the North Kivu Province of the Congo during an Ebola outbreak that she should take precautions against a virus she has never heard of, when two of her children recently died of malaria and the family is at constant risk of violence from the multitude of armed militias in the region – tragedies that seem to go unnoticed by the government and international response workers who have now flooded her town to confront a disease she cannot see? The complex socio-political, cultural, and economic antecedents of the outbreak or infectious disease du jour, so thoroughly explained by the late and gifted Paul Farmer in Fevers, Feuds, and Diamonds, about Ebola in Sierra Leone, were undeniable.^1^

### What exactly are human rights?

The idea of tropical medicine and human rights being intertwined is not exactly new. A review of ASTMH Presidents' Addresses over the last decade shows that most focused on activism more than science and research. Human rights were mentioned in four of the last five ASTMH Presidents’ Addresses, and I believe quite a few more before that.

What exactly are human rights? There are numerous documents and accords that seek to define them. The Universal Declaration of Human Rights was drafted by a committee chaired by Eleanor Roosevelt, U.S. President Franklin Delano Roosevelt’s wife, and adopted by the United Nations General Assembly in 1948.^2^ In its 30 articles, the document enshrines the rights and freedoms of all human beings, affirming their universal character as inherent, inalienable, and applicable to all. Someone must be paying attention, since the declaration holds the Guiness Book of World Records for the world’s most translated document, with over 500 translations.

**Figure 1.**
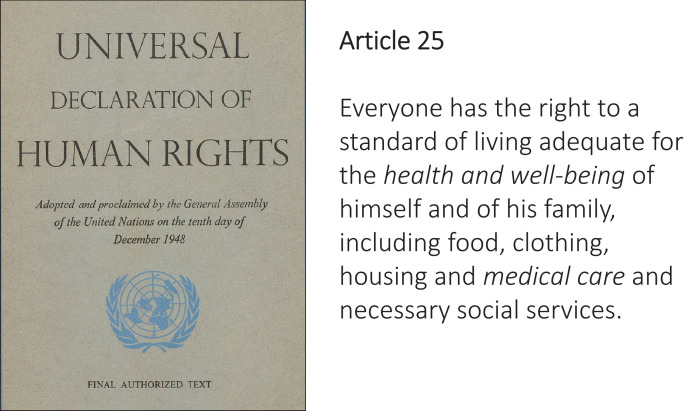
Article 25 of the Universal Declaration of Human Rights, which lays out health, well-being, and medical care as human rights.^2^

With specific regard to health, Article 25 stipulates that “Everyone has the right to a standard of living adequate for the health and well-being of himself and of his family, including food, clothing, housing, medical care, and necessary social services” (Figure 1). The UN further defined the right to health in Article 12 of the 1966 International Covenant on Economic, Social and Cultural Rights, recognizing “the right of everyone to the enjoyment of the highest attainable standard of physical and mental health.”^3^ The preamble of the 1946 WHO Constitution defines health broadly, as “a state of complete physical, mental, and social well-being, and not merely the absence of disease or infirmity”, enumerating some principles of this right as healthy child development; equitable dissemination of medical knowledge and its benefits; and government-provided social measures to ensure adequate health.^4^

But, of course, these are just documents and conventions. Do ASTMH members really require such detailed explanation? All you really need to do is ask yourself, “How would I like to be treated? What treatment of me would I claim is unjust? What care would I like to keep me healthy and restore my health if I fall sick”? I believe that your answers as to what constitutes a right to you, and to us all as humans, will be quickly evident.

### Human rights as a framework for tropical medicine and global health.

Why are human rights important as a framework for tropical medicine and for our Society? It is not to appear “woke” or to be on the perceived correct side of a political agenda, but rather because it is critical to our Society’s mission.

With our scientific contributions, the world has many positive results to note regarding health indicators. Life expectancies are drastically enhanced around much of the globe, many infectious diseases such as malaria and some neglected tropical diseases are under better control or even near elimination, and rates of protein-energy malnutrition have dropped, although many of these indicators took a step backward during the COVID-19 pandemic. Julie Jacobson’s Presidential Address last year detailed many of the positive health indicators and our Society’s role in bringing them about.^5^

Human rights are the right framework for tropical medicine, because, while the contribution of science to many of these health indicator gains is undeniable, I fear that in many domains we are reaching the limits of science’s power. As a result of our collective efforts, in many fields, we now have the right tools — diagnostics, therapeutics, vaccines — to do the job, but it nevertheless remains undone. We struggle with implementation of our science to ensure impact. The varied uptake of COVID-19 vaccines, so thoroughly addressed in Heidi Larson’s book “Stuck”, is the most obvious example to us all.^6^ I can also cite my time as the director of the United Kingdom Public Health Rapid Support Team (the United Kingdom’s entity for outbreak response in low- and middle-income countries), when very frequently the disease outbreak to which we were responding was vaccine-preventable. The outbreak had no business happening. The solution, the vaccine, already existed but had not reached the people in need.

Another reason to adopt a human rights framework is because rights are under attack. We tend to take them for granted. But I would venture to say, that in recent years there have been some concerns about our rights, that things have slipped when we let our guard down. Controversial computer intelligence consultant, Edward Snowden, put it simply, “Your rights matter, because you never know when you’re going to need them.”

Furthermore, the data are clear that pandemics and epidemics, occurring with increasing frequency, further widen inequities and human rights abuses. If we do not address inequities through a commitment to human rights, we will forever be behind—not only the wrong thing to do ethically, but a strategic mistake since, as COVID-19 taught us, we are all connected.

### Who ensures human rights?

Of course it is easy to say that something is a right, but that does not ensure its enactment. Indeed, human rights are so frequently trampled upon or ignored that upholding them can seem like a losing battle or waste of time. And for many human rights, there are few enforcement mechanisms.

Furthermore, the framework for human rights itself is far from perfect; the Universal Declaration was created largely by high-income western countries and is focused on individual as opposed to collective group rights. There are concerns over the over-arching reach of the State in assuring human rights. The language in the Universal Declaration is not gender neutral, referring consistently to he or him. Added to this is the question whether we in the field of “tropical medicine”, with its colonial and racist history, are best positioned to be champions of human rights.

Nevertheless, where would be without a framework of human rights? What standards would we use to assess right and wrong on the international stage, what justification to further our trade, to advocate for funding and access? I submit to you, that despite its imperfections and shortcomings, the Universal Declaration and its standards that have been developed over the last 75 years represent an incredible human achievement, expressing commonalities that we share, and at very least, provide a seed which sprouts recognition, and hopefully action, when rights are ignored.

As to who upholds human rights, Eleanor Roosevelt asked the same question, and answered this way, “Where, after all, do universal human rights begin? In small places, close to home — so close and so small that they cannot be seen on any maps of the world. Yet they are the world of the individual person; the neighborhood he lives in; the school or college he attends; the factory, farm or office where he works. Such are the places where every man, woman and child seeks equal justice, equal opportunity, equal dignity without discrimination. Unless these rights have meaning there, they have little meaning anywhere. Without concerned citizen action to uphold them close to home, we shall look in vain for progress in the larger world.”^7^

While Mrs. Roosevelt forgot to mention ASTMH as one of those local actors in 1948, as did then ASTMH President George Cheever Shattuck, I submit to you, that with regard to health, ASTMH is, and must be, that local embodiment, that neighborhood, that advocates for and upholds human rights, giving human rights real-world meaning through our work to make a healthier planet. We, as scientists and healthcare workers, must play our role as the advocates and guardians of the human right to health. Much of this work is direct through the evidence that researchers produce and care that clinicians and field workers provide. But we must also be the advocate, the reminder, and when necessary the thorn in the side, to politicians and policy makers to uphold this right. If we don’t perform this function, who will? Still further, we should consider the advice of former CDC Director and public health icon, Bill Foege, who encourages scientists and healthcare workers to enter the political realm themselves to advocate and set policy from within the political systems.^8^ Certainly, advocacy for health as a human right must be part of that job, and no group of people is more qualified to perform it than ASTMH members.

### Why ASTMH members should be ambassadors and advocates for human rights.

Why us, you may ask? Most of us consider ourselves first, and foremost, scientists and healthcare workers. Isn’t it the politicians’ and activists’ job to advocate for and ensure human rights? And, of course they must, but I can tell you we must also engage. Two reasons:

First, despite the troubling lack of trust in our society today, scientists, clinicians, and healthcare workers still enjoy a privileged vantage point in most societies around the globe. Justly or unjustly, this provides a podium to speak, to raise your voice, to be heard above the din. Furthermore, because your views and advocacy are evidence-based, you can make a better case that they are not politically motivated, not expressed because you are on the political left or right, but because the scientific evidence presents you with a truth that you can share.

Second, and most importantly, human rights advocacy falls within your job precisely *because* you are scientists and healthcare workers. And not only scientists, but life scientists. You did not choose to study paleontology or astronomy or to simply document the natural world. Your science is dedicated to intervention and change, to making lives happier, and healthier, and longer. Scientific pursuit, inherent in most of our daily lives, is a noble cause. But your mission is not simply pursuit of knowledge, but the pursuit of *health*. For us, science is a step in a process, not the end goal.

It is easy to become lost in your expertise, the disease that you study, the grant that you write, the paper that you publish, your struggle for tenure and recognition — all important things. But not truly yours, nor our Society’s, goal. Our job is not done, our goal not reached, when the scientific evidence is generated, the manuscript published (yes, ideally in some high impact journal to further our practical professional aims), unless that evidence, that paper, has impact—leading to a healthier person, patient, and planet.

### Don’t give in to pessimism or indifference.

I realize that some in the audience may feel frustrated, perhaps lamenting the days, if indeed they ever existed, when we and the Society “could just focus on the science.” With challenges seemingly coming from all sides, it is hard to stay optimistic. Nor can I claim any Pollyanna optimism. If you’re not alarmed by the present state of the world, you’re not paying attention.

Overwhelmed, we often continue our vertical focus on a particular disease — not wrong, but not wholly sufficient toward our goal of assuring health. No doubt, this is a protection against our feelings of helplessness about the suffering we often witness.

We do our best to make amends, through a “fix” to a part of the problem, but often know, inside, that we are ignoring the root cause.

I like to revert to two famous Paul’s for wisdom and stamina. The apostle Paul said, “I have fought the good fight, I have finished the race, I have kept the faith.” A few millennia later, another Paul, Paul Farmer, expressed a similar sentiment, albeit in a much different context, “We want to be on the winning team, but at the risk of turning our backs on the losers, no, it is not worth it. So we fight the long defeat.”

I take comfort in that whether we win or not, we fight the good fight, the long defeat, continuing to advance our scientific agendas and keeping our eyes on our goal of making humans and our planet healthier.

## THE SOCIETY’S COMMITMENT TO HUMAN RIGHTS AND CALL TO ACTION FOR ASTMH MEMBERS

I will conclude with expressing my personal, and through the privilege of serving as your president, the Society’s commitment to human rights. In the next year we will revisit the ASTMH strategic plan, into which I hope to codify human rights and to work with our Scientific Program Chair, Christy Peterson, to integrate and enhance human rights frameworks into our scientific program to ensure that they live beyond any one president’s tenure or any one Annual Meeting. This could include such offerings as a yearly lecture and symposium dedicated to health and human rights.

But, of course we have to do more. That is why I call on ASTMH members to make human rights the underpinning of your daily work. I believe, that recognized or not, human rights are why you chose this field, work so hard, and why you are a member of this Society sitting here today. This does not mean you must shut your laboratory, march in the street, or choose a new career. You do not have to quit your job and join Amnesty International or another organization solely dedicated to human rights. Rather, I propose five actions to incorporate into your ongoing work:

1. **Keep human rights, as well as Past President Julie Jacobson’s three Cs of compassion, culture, and courage, in all you do.**^5^ Do your science, study your protein conformation, your immune response, or see your patient, but keep human rights in mind. Talk about it with colleagues and students. Include a human rights framework in your lectures and writings to keep us from getting lost in the science, the proverbial forest for the trees. Human Rights Day, December 10th every year, can be a rallying moment.
2. **Be a witness and share your testimony.** Virtually all of you live or work in countries or settings, including in both low- and middle-income and high-income countries, where the right to health is often not respected or ensured. You see it regularly even if perhaps now it has become so routine that it ceases to shock. You are a witness to things that few politicians and policy makers can see. Your testimony, expressed in the right way, can bring problems to life, citing health problems and focusing politicians and policy makers in a way that cannot happen simply by reading a secondhand report.
3. **Advocate for universal health coverage and access to health services.** No matter what your field in the health sciences, it cannot have impact, and indeed becomes almost purposeless, unless a person’s right to healthcare is assured, allowing them to access the benefits your scientific work provides.
4. **Think about the value chain.** What will it take to maximize the impact of your work? No one person can do it all, but how can you link and support other collaborators and institutions, scientists and non-scientists, to assure that we address all the nodes along the value chain that result in maximum impact on health?
5. **Vote and advocate for human rights, within ASTMH and beyond.**

Your *means*, the thing you do every day, may be researcher or clinician, but your *end*, your end is improved health, in all its senses, and a fundamental respect and advocacy for human rights, is an essential tool to meet your goal.
